# Effect of Ramadan fasting on anthropometric parameters and food consumption in 276 type 2 diabetic obese women

**DOI:** 10.4103/0973-3930.53122

**Published:** 2009

**Authors:** Boumédiène Méghit Khaled, Slimane Belbraouet

**Affiliations:** Department of Biology, Faculty of Sciences, Djillali Liabès University, Sidi-Bel-abbès, Algeria; 1 Université de Moncton, Ecole des Sciences des Aliments, de Nutrition et d'Etudes Familiales (ESANEF), Moncton, Nouveau-Brunswick, E1A 3E9, Canada

**Keywords:** Food consumption, obesity, Ramadan fasting, Type 2 diabetes, weight

## Abstract

**AIM:**

To assess the effect of Ramadan fasting on body weight and food consumption in type 2 diabetic obese women.

**MATERIALS AND METHODS:**

A total of 276 outpatient women receiving oral antidiabetic drugs (OADs) (BMI = 34.63 ± 3.29 kg/m^2^), aged 49 (±6 years), were selected. The study was carried out over three periods - before (T1: prefasting), during (T2: fasting), and after (T3: postfasting) Ramadan - in three towns located in the northwestern region of Algeria. During the course of 3 days, the daily food intake and anthropometric parameters weight, height, waist and hip circumferences, body mass index (BMI), and waist-hip ratio (WHR) were recorded. A one-way repeated measures ANOVA test was used to compare the groups.

**RESULTS:**

The main effect of fasting during Ramadan was a significant weight loss (−3.12 kg i.e. 3.70%; *P* < 0.01), a decrease in meal frequency (2.2 ± 0.3 vs. 4.3 ± 0.4) as well as in energy intake (1488 ± 118 vs.1823 ± 262 Kcal/d) and an important increase in dietary fat consumption (35.84 vs. 25.36%), especially the saturated one (231Kca/d i.e. 43.25%) of total fat, dietary cholesterol (392 ± 121 vs. 221 ± 73 mg/d), and polyunsaturated fatty acids (PUFA). Except in three cases, there were no frequent hypoglycaemic episodes observed among the participants.

**CONCLUSIONS:**

Fasting during the month of Ramadan causes weight loss and decrease in calorie intake, which is correlated with a decrease in meal frequency. However, more foods rich in fat and dietary cholesterol were consumed during this period. The latter could constitute a high risk for diabetics who are fasting, in particular when medication advice and/or health care control are insufficient or ignored.

## Introduction

During the sacred month of Ramadan, people who fast neither eat nor drink from dawn to sunset. Among disabled individuals with acute or chronic diseases, certain diabetics can be exempted from fasting. Many people with diabetes still prefer to fast, without medical guidance, exposing themselves to certain health risks as a direct consequence of fasting or because of a change in food and frequency of medication intake. Although the benefit of experimental supplemented fasting has been well demonstrated, in diabetics, the consequential effects of fasting during Ramadan remain often controversial.[1–4] Modifying eating habits during Ramadan fasting and then significantly increasing one's intake after breaking the fast, could unbalance the metabolism of patients with diabetes and influence their nutritional intake and their anthropometric parameters.[5–13] Concerning diet during Ramadan, people usually eat two meals, one before dawn (Sahur) and one just after sunset (Iftar). Few studies have assessed the food intake among type 2 diabetics and we find conflicting results with those reported in.[14] A decrease in daily calorie intake has been seen as one of the advantages in Ramadan fasting.[5,11,17] Some authors[15] observed a decrease in energy intake (103 Kcal/d), though not statistically significant, which is correlated with meal frequency.[13] However, in another study,[18] the total daily energy intake (TEI) remained unchanged. Our study investigated the effect of fasting, during Ramadan, on some anthropometric parameters, and assessed food consumption among a group of obese women with type 2 diabetes mellitus (T2DM).

## Materials and Methods

### Patients and study design

The study was undertaken during Ramadan of 2004 in three towns located in the north-western region of Algeria (Saïda: Diabetes Centre, Sidi-Bel-Abbés: Petit-Vichy Diabetes Centre and University Hospital, and Oran: Hospital University). The volunteer outpatients who wanted to fast during Ramadan consisted of 276 type 2 diabetic obese women (BMI>30), aged 49 (±6) years, whose diabetes was identified 4 (±2) years ago and who presented no degenerative complications or hypertension. We focused our choice on diabetic and obese people because the prevalence of diabetes is primarily attributable to the rising rate of overweight and obesity.[19] Subjects were chosen if they had had diabetes for less than 5 years, because those who were preselected with over 5 years of diabetes presented some degenerative complications and, in this fact, were excluded. In addition, women were chosen for this study with the knowledge that another study would be done with male subjects. Throughout the study, diabetics received oral antidiabetic drugs (OADs); metformin: Glucophage^®^ 850mg, alone as monotherapy or associated with glimepiride: Amarel^®^ 3 or 4 mg as bitherapy. During fasting days, 160 patients were on single hypoglycaemic therapy (metformin) twice daily; the first one was taken at dawn and the second one at sunset (when fast was broken). The other 116 patients on bitherapy (metformin and glimepiride) once daily before the first meal and with the same dose, that is metformin 850 mg, glimepiride 3 or 4 mg. None of the diabetics, who gave their written consent following the explanation of the study protocol according to Djillali Liabés university ethics committee, followed any special restricted diet or smoked, but some guidance on how to manage medication during Ramadan was given. Physical activity, before and during Ramadan, was considered as moderate and restricted to housework for the majority of patients. Finally, women who were pregnant or breastfeeding were excluded from the study.

### Methods

The study lasted for three months and was scheduled over three periods: one month before Ramadan (T1: prefasting), during the month of Ramadan (T2: fasting at the middle), and one month after Ramadan (T3: postfasting). The protocol included a questionnaire in the form of an individual face-to-face interview. The objective of the interview was to gather sociodemographic data, current treatment, body weight status, lifestyle change during Ramadan and frequency of hypo or hyperglycaemia. Three measurements were taken and then averaged for weight and height. Body weight was measured, always in the morning, to the nearest 0.1 kg, with an electronic balance (SECA^®^-Germany; SECA 731 Sauna: Capacity: 150 Kg/Graduations: 1.000 g), with the participants wearing light clothing.

The BMI was defined as follows: BMI (kg/m^2^) = weight (kg)/height^2^ (m^2^). Waist-hip ratio (WHR) was determined by measuring the waist circumference (WC) at the narrowest part of the torso and the hip circumference in a horizontal plane at the level of the maximal extension of the buttocks. All anthropometric parameters were noted throughout the three periods.

The nutrient intake was evaluated by means of a 3-days food record. To this end, all of the patients were instructed on how to record their daily dietary intake. Food quantities were calculated using household measurements (plate, glass, slice, and bowl) during the three periods. Then individual records were reviewed, and the nutrient calculations were carried out using the USDA's food composition table.[20] Energy intake and diet composition nutrients were calculated with the Nutrinux v1.0 © software. Concerning metabolic control, blood samples were drawn after 12 h of overnight fasting and were collected over the three periods. The measure of fasting serum glucose (FSG), glycosylated haemoglobin (HbA1c), and lipid components (total cholesterol, triglycerides, HDL-cholesterol, LDL- cholesterol, apoA-I, and apoB) were used to present the biological results for the present study (data not shown).

### Statistical analysis

Values are expressed as mean ± SD. Analyses were performed using SPSS (version 15.0). Group means were compared using one-way repeated measures ANOVA test. Linear regression analysis was performed for determining the contribution of “Iftar” in the total energy intake (TEI). Additional correlations were made to determine the relationship between some parameters: weight, energy intake, and meal frequency. A *P-* value of less than 0.05 was considered as statistically significant.

## Results

### Anthropometric parameters

The main finding was a significant decrease (−3.12 kg i.e. 3.70%; *P* < 0.01) in body weight during T2 (81.14 ± 6.34 kg) when compared with T1 (84.26 ± 8.84 kg). However, this loss was not maintained, and patients regained about 2.45 kg (*P* < 0.02) one month later (T3 83.59 ± 7.21 kg). The circumference measurements, that is, WC and WHR, showed no significant difference during the three periods [Table 1].

**Table 1:** Anthropometric characteristics and nutrient intakes through the three periods in diabetic women. n=276

	T1	P value*	T2	*P* value#	T3	Dietary recommendations
Weight (kg)	84.26 ± 8.84	0.01	81.84 ± 8.67	0.01	83.60 ± 9.23	
BMI (Kg/m^2^)	35.16 ± 3.63	0.01	34.40 ± 3.64	0.01	35.14 ± 3.94	
WC (cm)	107.30 ± 8.06	NS	106.89 ± 7.96	NS	107.25 ± 8.01	
WHR	0.90 ± 0.06	NS	0.89 ± 0.05	NS	0.90 ± 0.05	
TEI. Kcal/d	1823 ± 262	< 0.001	1488 ± 118	< 0.001	1723 ± 227	1500 Kcal/d[23]
Carbohydrates g/d (%)	296 ± 78 (64.94)	< 0.001	193 ± 61 (51.88)	< 0.0001	271 ± 75 (62.91)	45-65%[37]
Dietary T bres g/d	18 ± 4	< 0.001	13 ± 3	< 0.001	16 ± 5	20-30 g/d[23,27]
Proteins g/d (%)	44 ± 11 (9.65)	< 0.001	46 ± 14 (12.37)	< 0.001	37 ± 10 (8.59)	15-20%[38]
Fats %	25.36	< 0.001	35.84	< 0.0001	28.57	< 25%[38]
SFA %	7.85	< 0.0001	15.50	< 0.0001	9.79	< 7%[37,39]
MUFA %	7.85	< 0.01	10.13	NS	8.71	> 20%[37]
PUFA %	9.42	NS	10.13	NS	10.34	∼10%[37]
LA (ω6) g/d	16 ± 3.4	NS	15 ± 2.3	< 0.01	17 ± 3,5	9-13g/d[21]
ALA(ω3) g/d	0.76 ± 0.08	< 0.001	0.69 ± 0.06	< 0.001	0.80 ± 0.08	1.4-2.6 g/d[40]
LA / ALA -	21.05	NS	21.74	NS	21.25	≤ 5[21]
TFA g/d	4.9 ± 1.2	< 0.01	6.3 ± 2.0	< 0.01	5.5 ± 1.4	< 2.8 g/d[41]
DHA(ω3) mg/d	86.2 ± 8.6	< 0.0001	72.3 ± 7.8	< 0.01	79.4 ± 7.2	120 mg/d[40]
EPA (ω3) mg/d	63.0 ± 6.5	< 0.01	58.4 ± 6.0	< 0.01	66.6 ± 6.9	500-800mg/d[21]
MUFA + Carbohydrates	72.72	< 0.001	61.45	< 0.001	71.26	60-70%[38]
Cholesterol mg/d	221 ± 73	< 0.0001	397 ± 124	< 0.0001	263 ± 82	< 300mg/d[37]

BMI; body mass index, WC; waist circumference, WHR; waist-hip ratio, Values between brackets in bold letter represent the percentage of calories with regard to the TEI. TEI, total energy intake; SFA, Saturated Fatty Acids; MUFA, Mono Unsaturated Fatty Acids; PUFA, Poly Unsaturated Fatty Acids,

* difference between T1 and T2;

# difference between T2 and T3.

### Food intake assessment

The significant decrease −335 Kcal/d (−18.38%: *P* < 0.0001) in TEI during T2 (1823 ± 262 *vs.* 1488 ± 118 Kcal/d), constituted the second finding of this study as shown in Table 2. The meal frequency also decreased during Ramadan when compared to T1 (2.2 ± 0.3 *vs.* 4.3 ± 0.4). The “Iftar” represented 76.49% of the TEI, while the “Sahur” counted only for 14.13% of the TEI. The dinner, which is taken a few hours after “Iftar” and usually after “Taraouih prayers” (religious prayer offered each night of Ramadan), represented just 2.08% of the TEI. As for the last meal “Sahur” it represented 14.13% of the total energy intake. Regarding nonfasting days, T1/T3, the majority of the daily calories were supplied by lunch (>35% of TEI) and dinner (∼ 30% of TEI).

**Table 2:** Total Energy Intake and the contribution of each meal during the three periods. n=276

	Meals	Kcal	%	Meals frequency
T1	TEI	1823 ± 262*§	100	
	Breakfast	182 ± 26	9.98	
	Collation	69 ± 16	3.79	
	Lunch	662 ± 88	36.31	4.3 ± 0.4§
	Snack	165 ± 27	9.05	
	Dinner	625 ± 85	34.28	
	Nibbling	120 ± 25	6.58	
T2	TEI	1488 ± 118#	100	
	Iftar†	1097 ± 361	73.72	
	Collation	18 ± 08	1.21	2.2 ± 0.3#
	Dinner	40 ± 12	2.69	
	Nibbling	92 ± 18‡	6.18	
	Sahur‡	241 ± 45	16.20	
T3	TEI	1723 ± 227§	100	
	Breakfast	224 ± 32	13.00	
	Collation	88 ± 13	5.11	
	Lunch	593 ± 85	34.42	4.4 ± 0.2§
	Snack	176 ± 24	10.22	
	Dinner	506 ± 69	29.37	
	Nibbling	136 ± 29	7.89	

* Data are given as mean ± Standard deviation.

† First meal (at sunset),

‡ Last meal (before sunrise). Significant difference between fasting

“#” and non fasting days

“§” (*P* < 0.001). TEI, total energy intake.

During T2, the nutrient intake, which is summarized in Table 1, showed a significant decrease (*P* < 0.001) in carbohydrate calorie consumption (T2: 193 ± 61 g/d, when compared to T1: 279 ± 80 g/d and T3: 259 ± 75 g/d). The dietary fiber intake decreased significantly during T2: (13 ± 3 g/d) when compared to T1: (18 ± 4 g/d) and T3: (16 ± 5 g/d; *P* < 0.001), though the protein intake increased with regard to T1: (49 ± 15 *vs.* 42 ± 12; *P* < 0.001). On one hand, Ramadan diet increased the dietary fat intake (*P* < 0.0001) to 35.84% of TEI when compared to T1: 25.36%. On the other hand, it not only brought fatty foods into the diet, but also saturated fatty acids to 15.50% of TEI (i.e. 43.23% of the total fat calories). The amount of carbohydrates plus monounsaturated fatty acids (MUFA) during T2 decreased significantly (62.01%; *P* < 0.001) when compared with the other periods: 72.79% for T1 and 71.62% for T3.

Regarding the polyunsaturated fatty acids (PUFA), the consumption of linoleic acid (LA) was important during all of the periods and exceeded the dietary recommendations (9-13 g/d[21]). However, the alpha-linolenic acid (ALA) consumption was poor during Ramadan (0.69 ± 0.06 g/d) when compared to the other periods (T2: 0.76 ± 0.08) and (T3: 0.80 ± 0.08; *P*<0.001), and the LA/ALA ratio during all periods was also high. Concerning the other omega-3 fatty acids, such us Docosahexaenoic acid (DHA) and Eicosapentaenoic acid (EPA), their intake, during T2, was very limited, particularly for the EPA: 58.4 ± 6.0 g/d when compare with dietary recommendations (500–800 mg/d). Moreover, diet during Ramadan brought more *trans* fatty acids (TFA) (6.3 ± 2.0 g/d) and more dietary cholesterol (397 ± 124 mg/d) as shown in Table 1, all of which exceeded the dietary recommendations.

Concerning the metabolic profile, Ramadan fasting induces a significant decrease of FSG: (9.26 ± 2.87 *vs.* 7.32 ± 1.22 mmol/l; *p<0.001)* and HbA1c: (9.17 ± 1.62 *vs.* 8.14 ± 0.90 %; *p<0.001)*, which was maintained one month later. For the lipid components, a major change has been seen with an increase in total cholesterol (TC) rate: (7.82 ± 1.74 *vs.* 5.18 ± 1.34 mmol/l; *p<0.001)*. These values fell during T3. The principal factor consisted in SFA, which contributed to raise the LDL-c: 3.46 ± 0.73 mmol/l and TC concentrations. This increase was associated with a decrease of body weight, particularly for TC that was significantly correlated with weight loss (*r*^2^=−0.318; *p*<0.01).

### Drug intake

We did not observe any frequent hypoglycaemic episodes in our diabetic patients treated with metformin and/or glimepiride, except for three cases: two patients took their medication without eating “Sahur” and one expended an important amount of physical energy.

## Discussion

Obesity and weight gain are considered to be among the most substantial risk factors for developing T2DM.[22] In diabetic obese individuals, weight gain deserves high priority because about 80% of people with T2DM are overweight or have abdominal obesity. Weight loss during Ramadan constitutes the main beneficial effect of fasting in our diabetics, and was correlated with the decrease in energy intake (*r*^2^=0.180, *P* < 0.01). However, unfortunately a regain of 2.45 kg was observed in T3 and was linked both to an increase in calorie intake (*r*^2^=0.177, *P* < 0.01) and to diet mismanagement. It has been shown that a loss of about 4.5 to 9 kg is helpful and should be maintained on a long-term basis to avoid rapid regaining of weight pre fasting level.[23] According to Azizi and Siahkolah,[14] overweight subjects lose more weight, during Ramadan, than those deemed normal or underweight. The other anthropometric parameters (WC, WHR) showed no significant difference throughout the three periods.

**Figure 1:**
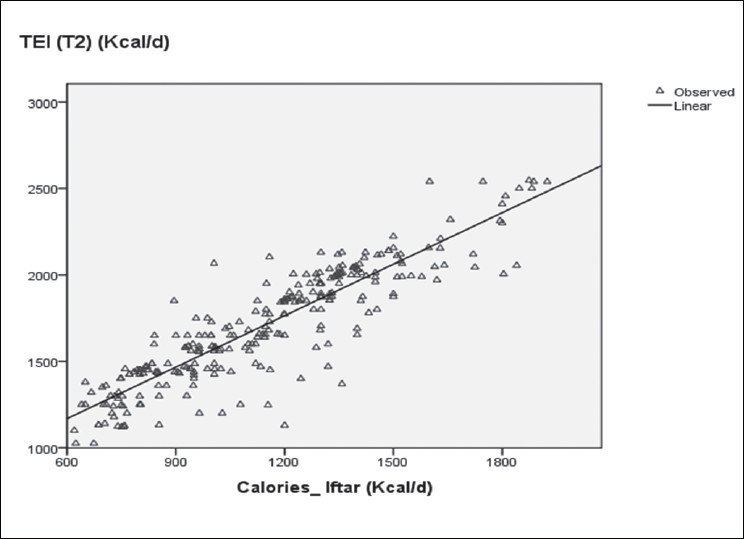
Contribution of calories brought by “Iftar” in the daily TEI during Ramadan TEI Ramadan: Total energy intake during Ramadan. Calories_Iftar: calories brought by “Iftar” meal

Concerning the Ramadan diet, literature reveals that meal frequency decreases in healthy people as well as in diabetics. In our patients, we noticed a decrease in the number of meals that significantly contributed to reduce the amount of calories (*r*^2^=0.412, *P* < 0.001). “Iftar” is the main meal during this period and increases substantially the TEI (*r*^2^=0.802), as illustrated in Figure 1, due to the change in eating habits among nearly all patients who preferred taking their meal one at a time, while others preferred having soup or a glass of milk and the rest of the meal would be eaten after practicing “Taraouih” prayers. These eating habits depended on place and family customs. The total calories brought by “Iftar”, nibbling, and dinner represented about 80% of the TEI and were consumed within a very limited amount of time in the evening (∼ 4–5 hours interval). “Sahur” brought only 14.13% of energy calories, because several patients (31%) were not used to waking up early (dawn) and skipped the meal. However, this later is very important, because it enables diabetic fasters to avoid hypoglycaemic discomforts throughout the fasting day, particularly for patients observed their medical prescription before undertaking the fasting. The Ramadan fasting diet appears to be a good opportunity to reduce calorie intake (−335 kcal/d) caused by a reduction in meal frequency. During nonfasting days (T1 and T3), some patients try to skip some meals in order to lose a few kilograms, like breakfast, and as a result calories of lunch increased to 37.20 % of TEI for T1and to 35.05 % of TEI for T3.

In patients with diabetes, no clear-cut formula exists for the three nutrient intakes (carbohydrate, fat, and protein), and the key is that the calorie intake will be relative to the calorie output. The contribution of carbohydrates and monounsaturated fats should be individualized and based on nutritional assessment, metabolic profile, and treatment goals.[24] Currently, the average amount of carbohydrates recommended for patients with diabetes is 55–60% of the TEI, and little evidence is available to support the belief that sugar should be avoided or that starch should be favoured.[25] Therefore, in agreement with the National Academy of Sciences - Food and Nutrition Board,[26] the recommended range of carbohydrate intake is 45–65% of TEI. Ramadan fasting induces a significant decrease (*P* < 0.001) in carbohydrates, because obese women who want to improve their serum glucose and reduce calories, refrain from eating sweet or starchy foods, following their physician's advice. Consequently, as we have observed, they consumed more fatty foods, thus increasing their consumption during Ramadan (35.84% of TEI), which exceeds dietary recommendations and conventional therapy values for treating obesity (25-30%).[23] The current dietary recommendations recognize that the total amount of carbohydrates is more important than its type, and diets with very low amounts of this nutrient tend to be high in fat and are therefore associated with weight gain and heart disease.[27]

More saturated fatty acids (15.50% of TEI) and dietary cholesterol (397 ± 124 mg/d) were brought during Ramadan. This was due to the foods rich in these nutrients, such as eggs, meat, chicken, fried food, various salads with mayonnaise, dairy fats, and shortening, which were highly consumed during Ramadan, whereas the dietary guidance for diabetics recommends that <7% of SFA is derived from saturated fats.[23] Regarding the MUFA, we find considerable solid scientific support in literature regarding their role in the treatment and prevention of T2DM,[28,29] but quantities that are consumed throughout the three periods are still insufficient. In the region, that we studied (Sidi Bel-Abbes and Saida), food rich in MUFA were poorly consumed, except for the town of Oran located on Algerian coast and where fish and seafood are more readily available. Owing to the excessive use of vegetable oils, such as sunflower oil or margarine, the amounts of PUFA represented by LA of 15 ± 2.3g/d - which accounted for about 80% of the total polyunsaturated fatty acids, and TFA of 6.3 ± 2.0 g/d, were higher during Ramadan than other periods [Table 1]. Concerning the omega-3 fatty acids that are considered as beneficial for diabetics,[30] their consumption was not sufficient, especially for the EPA. The same finding was observed for the ALA throughout the three periods [Table 1]. This was probably caused by the fact that the major sources of this nutrient, which are flaxseed, soy bean, canola, wheat germ, or walnuts oils, are not generally consumed in Algeria. The high reported values of LA/ALA ratio (> 21) showed a significant consumption of LA against ALA. Similar to the general population, intake of proteins, in type 2 diabetics, should be 10–20% of the TEI. Ramadan diet brought more food rich in proteins when compared with other periods. This is the case, because various dishes based on meat, dairy product, and fruits were consumed in greater numbers during this month.

Interview results suggested that physical activity was considered as moderate and was restricted to housework and walking, which is the most popular form of activity for the majority of patients, and we did not observe any significant change in the investigated periods. Still, during Ramadan daytime, some women feared hypoglycaemia and deliberately decreased their physical activity by sleeping or watching television. These findings are in accordance with those obtained by the EPIDIAR study[31] carried out in 13 Muslim countries over 11000 patients with T2DM, whose daily physical activity was considered as light to moderate for 94% of the studied population and remained unchanged in approximately half of them.

Concerning the drug intake during Ramadan, no signs or symptoms of hypoglycaemia were observed among the diabetic women participating in the study, except for three cases; two patients took their medication (Glimepiride 4mg) and ate nothing at dawn, “Sahur”, and the third undertook strenuous physical effort during the daytime. We noticed that, in the last cited study and in one-fourth of patients under oral treatment, the change in OADs doses by diabetics induced severe hypoglycaemic symptoms.[31] Some authors suggest that the modification of lifestyle and food intakes, which occur during Ramadan, should take into account an appropriate OAD.[32] The GLIRA (Glimepiride in Ramadan) study[33] and recent findings of Anwar et al,[34] highlighted the beneficial effect of Glimepiride with its longer period of action among patients with T2DM during Ramadan. According to Shaik et al[35] and the ADA work group report,[36] diabetics with obesity who take Metformin, are at minimal risk of hypoglycaemia. Otherwise, we did not observe any difference in the frequency of hypoglycaemia between the group taking Metformin and the group being administered bitherapy (i.e. Metformin plus Glimepiride). Finally, we should say that for our diabetics, Ramadan fasting constituted neither inconvenience nor discomfort and on the contrary they felt healthy when they worshipped and took precautions for their diabetes simulataniously.

## Conclusion

For obese people and particularly for diabetics, Ramadan fasting represents an excellent opportunity to initiate healthy lifestyle changes and to lose weight. It gives a real motivation for self management, which is highly needed during this period of the year. Moreover, the change in eating patterns, which occurs during this month, breaks previous habits. Nevertheless, when managing diabetes during this period, other points should be considered for diabetics whose physicians deem capable of fasting - quality of diet, physical activity, and choice of OAD- because it is often said that the beneficial effects of fasting during Ramadan will occur only in the patients who maintain a diet that is appropriate to them. Ramadan fasting induces weight loss, which was correlated with a decrease in the number of meals and calorie intake. Unfortunately, this weight loss was not maintained one month after the end of the fasting period, due to the mismanagement of eating habits and lifestyle. The TEI decreases during Ramadan, whereas the dietary fat consumption increases because of an augmentation of fatty food that does not occur during other periods (T1 and T3), as well as saturated food with a high amount of TFA, LA, and cholesterol due to the excessive use of vegetable oil and the high consumption of fried food. As reported by the ADA work group report,[36] hypoglycaemia is less frequent in patients with T2DM than type 1 diabetes and with less severe consequences.

These findings show that there is no contraindication for T2DM to fast during Ramadan. Only three subjects developed hypoglycaemic episodes. If appropriate instructions concerning diet and medication regimen are followed, this will not happen. For diabetics who wish to fast safely, diet modification remains a frontline strategy for controlling diabetes during this period. The medication regimen needs to be modified in timing and possibly in dosage, and should be adapted to the needs of individual patients.
